# Comparative Efficacy of Angiotensin Converting Enzyme Inhibitors and Angiotensin Receptor Blockers after Coronary Artery Bypass Grafting

**DOI:** 10.1038/s41598-020-58705-0

**Published:** 2020-02-03

**Authors:** Jeayoun Kim, Jungchan Park, Jong-Hwan Lee, Jeong Jin Min, Seung-Hwa Lee, Young Tak Lee, Wook Sung Kim, Sanghoon Song, Jung Hyun Yeo, Hyojin Cho

**Affiliations:** 1 Department of Anesthesiology and Pain Medicine, Samsung Medical Center, Sungkyunkwan University School of Medicine, Seoul, Korea; 2 Division of Cardiology, Department of Medicine, Heart Vascular Stroke Institute, Samsung Medical Center, Sungkyunkwan University School of Medicine, Seoul, Korea; 3 Department of Thoracic and Cardiovascular Surgery, Samsung Medical Center, Sungkyunkwan University School of Medicine, Seoul, Korea; 4 Department of Anesthesiology and Pain Medicine, Soonchunhyang University Seoul Hospital, Seoul, Korea

**Keywords:** Cardiology, Medical research

## Abstract

Although angiotensin receptor blockers (ARBs) are considered as an alternative for those with angiotensin converting enzyme inhibitors (ACEi) intolerance, the comparative effectiveness of ARBs and ACEi remains controversial in patients who underwent coronary artery bypass grafting (CABG). We aimed to compare the clinical effects of the two types of renin-angiotensin-aldosterone system (RAAS) inhibitors in patients who underwent CABG. From January 2001 to January 2015, among the 5456 patients, data from 1198 (20.1%) patients who used a RAAS inhibitor at discharge were analyzed. These 1198 patients were classified into ACEi (N = 900) and ARB (N = 298) groups. The primary outcome was major adverse cardiovascular and cerebrovascular events (MACCE) during a median follow-up period of 48 months. Propensity-matched analysis revealed that the incidence of MACCE over a 48 month follow-up period did not differ between the groups (HR, 0.65; 95% CI, 0.36–1.21; p = 0.17), but it was significantly lower in the ARB group during the 12 month follow-up period (HR, 0.46; 95% CI, 0.22–0.96; p = 0.04). In conclusion, ARBs may have comparable protective effects to ACEi and be a reasonable alternative for intolerant patients after CABG. The beneficial effects of ARBs depending on follow-up period require further investigation.

## Introduction

Secondary prevention is an integral part of ischemic heart disease treatment and also maximizes the clinical benefits of coronary artery bypass grafting (CABG)^1^. Renin-angiotensin-aldosterone system (RAAS) inhibitors have a cardioprotective effect by inhibiting angiotensin II, a potent vasoconstrictor that reduces renal perfusion and stimulates left ventricular hypertrophy, cardiac remodeling, and arterial hyperplasia^2^. However, there is still a debate on the comparative effects of the two discrete types of RAAS inhibitors (angiotensin converting enzyme inhibitors [ACEi] and angiotensin receptor blockers [ARBs]).

Current guidelines on ischemic heart disease suggest ACEi as the primary choice for secondary prevention of ischemic heart disease, and ARBs are considered as an alternative for those with ACEi intolerance^3–5^. That is because unlike the ACEi, which has shown relatively well-established cardioprotective effects, the clinical trials of ARBs for secondary prevention have shown inconsistent results in previous studies, especially in subgroups of patients with diabetes mellitus^6^, hypertension, or a history of myocardial infarction (MI)^7–13^. The effects of the two types of RAAS inhibitors have also not been compared in CABG patients. Therefore, in this study, we aimed to compare the effects of RAAS inhibitors by comparing clinical outcomes after CABG in patients prescribed postoperative ACEi or ARB therapy. Our findings might help select the type of RAAS inhibitors in secondary prevention after CABG.

## Results

Among 5,453 consecutive CABG patients, 74 patients were not prescribed discharge medication because of in-hospital mortality. After excluding patients without a prescription of RAAS inhibitors (N = 4,158) or with concomitant prescription of RAAS inhibitors (N = 23), a total of 1,198 patients were finally left for analysis and were classified into the two groups (ACEi group [N = 900, 75.2%] and ARB group [N = 298, 24.8%]). During the first year after CABG, discontinuations of RAAS inhibitors were found in 4 (1.3%) patients of the ARB group and 11 patients (1.2%) of the ACEi group. Changes to another type of RAAS inhibitors were found in 2 (0.7%) patients in the ARB group to ACEi and 101 (11.2%) patients in the ACEi group to ARB.

### Patient characteristics

Preoperative variables of the entire population are summarized in Table 1. Compared with the ACEi group, patients in the ARB group were older, more likely to have hypertension, diabetes mellitus, chronic kidney disease, and/or peripheral artery occlusive disease. Cardiopulmonary bypass was more frequent in the ACEi group. The ACEi group tended to have decreased ejection fraction below 40% and had a higher prevalence of old MI. After performing propensity score matching, a matched data set of 298 pairs was generated by 1:1 individual matching without replacement. There was no significant imbalance in baseline variables between the two groups of the matched population (Table 1).

**Table 1:** Baseline characteristics of entire and propensity-score-matched populations.

	Entire population				Propensity matched population		
	ARB group (N = 298)	ACEi group (N = 900)	p-value	SMD	ARB group (N = 298)	ACEi group (N = 298)	SMD
Male	193 (64.77)	614 (68.22)	0.29	0.07	183 (63.54)	183 (63.54)	0
Age	65.93 (±8.8)	63.90 (±9.3)	0.001	0.22	65.78 (±8.78)	66.12 (±8.08)	0.04
Diabetes	184 (61.74)	445 (49.44)	<0.001	0.25	176 (61.11)	174 (60.42)	0.01
Hypertension	240 (80.54)	578 (64.22)	<0.001	0.37	231 (80.21)	233 (80.90)	0.02
Dyslipidemia	107 (35.91)	279 (31.00)	0.12	0.1	103 (35.76)	104 (36.11)	0.01
Chronic kidney disease	45 (15.10)	44 (4.89)	<0.001	0.35	35 (12.15)	30 (10.42)	0.06
Stroke	54 (18.12)	133 (14.78)	0.17	0.09	50 (17.36)	46 (15.97)	0.04
Chronic obstructive pulmonary disease	5 (1.68)	27 (3.00)	0.22	0.09	5 (1.74)	4 (1.39)	0.03
Peripheral artery disease	42 (14.09)	76 (8.44)	0.005	0.18	35 (12.15)	42 (14.58)	0.07
LMD	46 (15.44)	149 (16.56)	0.65	0.03	45 (15.63)	49 (17.01)	0.04
3VD	212 (71.14)	633 (70.33)	0.79	0.02	204 (70.83)	207 (71.88)	0.02
Ejection fraction <40%	55 (18.46)	254 (28.22)	0.01	0.23	54 (18.75)	50 (17.36)	0.04
History of MI	32 (10.74)	160 (17.78)	0.004	0.2	31 (10.76)	32 (11.11)	0.01
history of PCI	50 (16.78)	174 (19.33)	0.33	0.07	49 (17.01)	49 (17.01)	0
CABG for Acute coronary syndrome	152 (51.01)	484 (53.78)	0.41	0.06	146 (50.69)	133 (46.18)	0.09
*Medication at discharge*							
Beta blocker	223 (74.83)	618 (68.67)	0.04	0.14	216 (75.00)	206 (71.53)	0.08
Antiplatelet	37 (12.42)	125 (13.89)	0.52	0.04	274 (95.14)	271 (94.10)	0.05
Calcium channel blocker	106 (35.57)	281 (31.22)	0.16	0.09	102 (35.42)	110 (38.19)	0.06
Statin	227 (76.17)	680 (75.56)	0.83	0.01	221 (76.74)	230 (79.86)	0.08
*Procedural character*							
Emergency operation	23 (7.72)	61 (6.78)	0.58	0.04	22 (7.64)	22 (7.64)	0
Redo-operation	2 (0.67)	21 (2.33)	0.07	0.14	2 (0.69)	1 (0.35)	0.05
Off pump CABG	240 (80.54)	636 (70.67)	<0.001	0.23	233 (80.90)	235 (81.60)	0.02
Artery graft	67 (22.48)	243 (27.00)	0.12	0.11	64 (22.22)	69 (23.96)	0.04
Valve combined operation	23 (7.72)	84 (9.33)	0.4	0.06	22 (7.64)	19 (6.60)	0.04

Values are N (%) or mean (±standard deviation).

ARB, Angiotensin receptor blocker; ACEi, Angiotensin converting enzyme inhibitor; N; Number, SMD, Standardized mean difference; LMD, Left main coronary artery disease; 3VD, Three vessel coronary disease; MI, Myocardial infarction; PCI, Percutaneous coronary intervention; CABG, Coronary artery bypass grafting.

### Clinical outcomes

**Figure 1:**
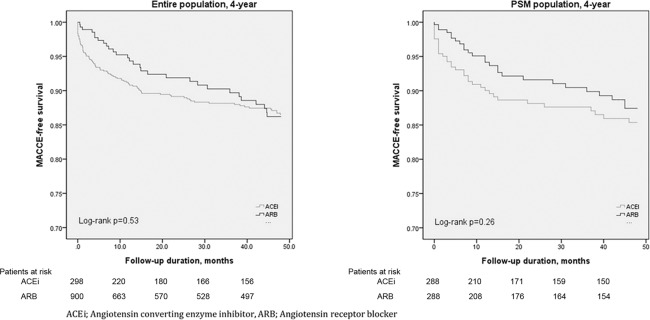
Kaplan-Meier survival curve analysis for major adverse cardiovascular and cerebrovascular events (MACCE) in the ARB group versus the ACEi group in the propensity-score-matched population and overall population over a 4-year follow up.

The median follow-up durations were 61.7 months (interquartile range: 8–107.9) in the ACEi group and 45.3 months (interquartile range: 8.6–86.2) in the ARB group (p = 0.66). Kaplan-Meier curves of the entire and propensity-matched populations are shown in Fig. 1.

Table 2 shows the cumulative incidences of clinical outcomes of the entire population. Multivariate Cox’s proportion hazard regression analysis revealed that the incidence of major adverse cardiovascular and cerebrovascular event (MACCE) during 48 months of follow-up did not differ between the two groups (hazard ratio [HR], 0.69; 95% confidence interval [CI], 0.44–1.06; p = 0.09), but the incidence was significantly lower in the ARB group at the 12 month follow-up (HR, 0.49; 95% CI, 0.27–0.91; p = 0.02). Similar results were obtained in the propensity-matched analysis (HR, 0.65; 95% CI, 0.36–1.21; p = 0.17 for the 48 month follow-up and HR, 0.46; CI 95% 0.22–0.96; p = 0.04 for the 12 month follow-up) (Table 3). In the analysis after excluding patients who discontinued or changed the class of RAAS inhibitors during the first year after CABG, the clinical outcomes did not differ between the two groups (Supplementary Table 1). The numbers of adverse events such as MACCE and cancer per 1000 months were also provided in Supplementary Table 2.

**Table 2:** Incidence rate and adjusted hazard ratio of clinical outcomes in entire population.

N, (%)	ARB group (N = 298)	ACEI group (N = 900)	Hazard Ratio (95% CI)	p-value
*4-year follow up*				
MACCE	29 (9.7)	101 (11.2)	0.69 (0.44–1.06)	0.09
Total death	12 (4.0)	26 (2.9)	0.57 (0.25–1.30)	0.71
Cardiac death	4 (1.3)	8 (0.9)	1.48 (0.39–5.73)	0.57
MI	5 (1.7)	12 (1.3)	0.80 (0.26–2.50)	0.7
Re-revascularization	7 (2.3)	37 (4.1)	1.15 (0.54–2.46)	0.18
Stroke	10 (3.4)	38 (4.2)	0.57 (0.28–1.19)	0.14
Heart failure	156 (52.3)	384 (42.7)	0.80 (0.26–2.50)	0.11
Graft failure	7 (2.3)	28 (3.1)	0.57 (0.25–1.30)	0.92
*1-year follow up*				
MACCE	13 (4.4)	73 (8.1)	0.49 (0.27–0.91)	0.02
Total death	4 (1.3)	9 (1.0)	1.42 (0.38–5.33)	0.6
Cardiac death	3 (1.0)	3 (0.3)	5.55 (0.75–41.0)	0.09
MI	1 (0.3)	8 (0.9)	0.29 (0.03–2.52)	0.26
Re-revascularization	3 (1.0)	28 (3.1)	0.36 (0.11–0.20)	0.1
Stroke	5 (1.7)	32 (3.6)	0.41 (0.15–1.07)	0.07
Heart failure	3 (1.0)	1 (0.1)	32.43 (0.94–1122.4)	0.05
Graft failure	7 (2.3)	17 (1.9)	1.61 (0.64–4.04)	0.31

Values are N (%).

ARB, Angiotensin receptor blocker; ACEi, Angiotensin converting enzyme inhibitor; CI, Confidence interval; MACCE, Major adverse cardiovascular and cerebrovascular events and composite of total death, cardiac death, myocardial infarction, re-revascularization and stroke; MI, Myocardial infarction.

Cox hazard model regression analysis was adjusted for Age, Sex, diabetes mellitus, hypertension, left ventricular ejection fraction <40%, chronic renal failure, peripheral artery disease, old myocardial infarction, beta-blocker therapy, off-pump coronary artery bypass grafting.

**Table 3:** Incidence rate and hazard ratio of clinical outcomes in propensity-score-matched population.

N, (%)	ARB group (N = 298)	ACEi group (N = 298)	Hazard Ratio (95% CI)	p-value
*4-year follow up*				
MACCE	26 (9.6)	35 (12.2)	0.65 (0.36–1.21)	0.17
Total death	11 (3.8)	8 (2.8)	1.17 (0.39–3.47)	0.78
Cardiac death	4 (1.4)	1 (0.3)	3 (0.31–28.84)	0.34
MI	5 (1.7)	3 (1.0)	2 (0.37–10.92)	0.42
Re-revascularization	7 (2.4)	12 (4.2)	0.78 (0.29–2.10)	0.62
Stroke	8 (2.8)	16 (5.6)	0.50 (0.19–0.33)	0.17
Heart failure	151 (52.4)	129 (44.8)	1.14 (0.87–1.48)	0.35
Graft failure	7 (2.4)	6 (2.1)	1 (0.32–3.10)	1
*1-year follow up*				
MACCE	13 (4.5)	25 (8.7)	0.46 (0.22–0.96)	0.04
Total death	4 (1.4)	2 (0.7)	4 (0.45–35.79)	0.22
Cardiac death	3 (1.0)	0 (0.0)	7 (0.23–214.37)	0.19
MI	1 (0.3)	2 (0.7)	0.50 (0.05–5.51)	0.57
Re-revascularization	3 (1.0)	9 (3.1)	0.38 (0.10–1.41)	0.15
Stroke	5 (1.7)	14 (4.9)	0.42 (0.14–1.18)	0.1
Heart failure	3 (1.3)	0 (0.0)	7 (0.23–214.37)	0.27
Graft failure	7 (2.4)	4 (1.4)	1.5 (0.42–5.32)	0.53

Values are N (%).

ARB, Angiotensin receptor blocker; ACEi, Angiotensin converting enzyme inhibitor; CI, Confidence interval; MACCE, Major adverse cardiovascular and cerebrovascular events and composite of total death, cardiac death, myocardial infarction, re-revascularization and stroke; MI, Myocardial infarction.

**Figure 2:**
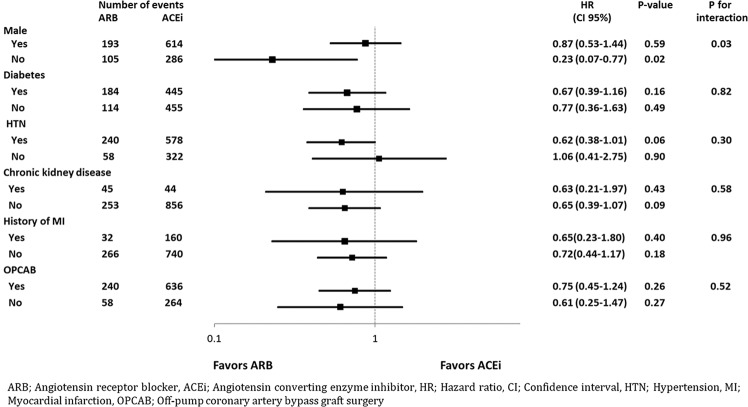
Forest plots from subgroup analysis showing the associations between risk of major adverse cardiovascular and cerebrovascular events (MACCE). Multivariate Cox proportional hazard analyses were used to evaluate risk factors associated with risk of MACCE. Horizontal lines are 95% confidence intervals (CIs) for the hazard ratios (HRs).

Subgroup analysis showed a significant interaction between sex and the use of RAAS inhibitors on the primary outcome. The use of ARB was beneficial only in female (HR, 0.23; 95% CI, 0.07–0.77, p = 0.02). Except for sex, there was no interaction between the various covariates and the use of RAAS inhibitors. Results of the subgroup analysis are shown in the hazard-ratio forest plots in Fig. 2.

## Discussion

The main findings of the present study were as follows: (1) postoperative use of ARB after CABG showed a comparable clinical effect to ACEi during median follow-up period of 48 months; (2) the use of ARBs resulted in a significant reduction in adverse events in terms of MACCE during 12 months of follow-up; (3) ARBs showed improved clinical outcomes limited to female patients. These findings suggest that in patients who underwent CABG, the effects of the two discrete types of RAAS inhibitors may be comparable, but can differ according to follow-up period or in particular subgroups.

ACEi is the most widely prescribed type of RAAS inhibitors because of well-established evidence of their cardioprotective effects by suppressing of renin-angiotensin-aldosterone system activity, resulting in reduced mortality^2^. On the other hand, the clinical effects of ARBs for secondary prevention of ischemic heart disease are relatively unclear especially in particular subgroups of patients^6,12,13^. The recent CABG guidelines also recommend ACEi as the primary choice of RAAS inhibitor, especially when MI, LV dysfunction, diabetes mellitus, or chronic kidney disease is present^1^. However, up to 20% of patients reportedly show adverse reactions towards ACEi, such as the development of cough and angioedema, and according to the guidelines, ARBs should be considered as alternatives to ACEi in these patients^3–5,14^. Moreover, in Asian populations, the prevalence of cough induced by ACEi is higher compared to Caucasian populations^15^. Therefore, the withdrawal rate of ACEi is high, and the prescription of ARBs is relatively more common in Asian populations^15^. Our data also showed 11.2% of withdrawal rate. In this context, understanding the comparative effects of the two discrete RAAS inhibitors would be helpful for clinicians.

In this study, postoperative use of ARBs did not result in a different clinical outcome in patients who had undergone CABG relative to those prescribed ACEi during a median follow-up period of 48 months. This result is consistent with that of a recent meta-analysis of randomized trials comparing the effects of ARBs to ACEi in terms of MACCE in patients with heart failure (HF)^7^. However, previous results were inconsistent in other subgroups of patients with higher risk. While the OPTIMAAL (Optimal Trial in Myocardial Infarction with the Angiotensin II Antagonist Losartan) trial could not prove either ‘superiority’ or ‘non-inferiority’ of ARBs relative to ACEi^16^, a more recent study showed that the use of ARBs lowered the rates of cardiovascular events compared to ACEi^17^. The largest randomized controlled trial conducted to date also reported no significant difference in cardiovascular outcomes between the two types of RAAS inhibitors in patients at high risk for vascular events^18^.

Intriguingly, our results showed that ARBs may be more beneficial than ACEi under particular conditions. The incidence of MACCE was reduced in the ARB group during 12 months of follow-up, and based on numeric data, this reduction seemed to be mainly driven by the stroke prevention effect of ARB. Experimental evidence has shown that ARBs can prevent stroke by blocking the local angiotensin II type 1 receptor and simultaneously allowing angiotensin II to stimulate unoccupied angiotensin II type 2 receptors, increasing local blood flow to the brain and preventing the death of injured cells in ischemic areas^19^. In addition to experimental evidence, a recent meta-analysis also demonstrated a meaningful stroke prevention effect of ARBs in comparison to ACEi^20^. However, the benefit of ARBs over ACEi vanished to the level of insignificance during 48 months of follow-up. This could be explained by ‘aldosterone-breakthrough’, which refers to the phenomenon whereby when RAAS inhibitors are used long-term, aldosterone levels are restored to baseline levels or reach even higher levels after an initial decline. It has been also reported that suppression of the RAAS is reversed in 10–53% of patients after 1 year of administration^21^. Considering that aldosterone is the end product of the RAAS and that it promotes tissue inflammation and injury in the cardiovascular and renal systems, this phenomenon explains the disappearance of the beneficial effect of ARBs compared to ACEi after a certain period of drug administration. Subgroup analysis revealed that the beneficial effects of ARBs persisted during 48-months of follow-up in female patients. This may be related to sex-based differences in the effectiveness of RAAS inhibitors, but needed further investigations^22,23^. In addition, the two types of RAAS inhibitors have shown discrete effects on other health issues such as cancer or infection^24,25^. However, these effects did not show a significant difference in our analysis.

Based on the results of this study, ARBs have a comparable effect to ACEi for secondary prevention after CABG, and therefore prescription of an ARB as an alternative to an ACEi seems reasonable for intolerant patients. In addition, ARBs may be considered as a primary choice for patients with a risk of stroke during the initial period after CABG. Our results also suggest that the gender of the patient might also alter the effects of RAAS inhibitor. However, well-designed further studies are needed to confirm our findings.

This study has several limitations. First, as a single-center retrospective study, unmeasured factors might have affected the results despite our efforts to adjust for all confounding factors by propensity score matching and multivariate logistic regression analysis. Second, the complexity of the procedure and post-operative care might have impacted clinical outcomes. Although we followed our institutional protocols, they have been updated during the course of our long study period. Lastly, types of ACEi or ARBs were not specified. Different types of RAAS inhibitors might have resulted in different outcomes. In addition, the dose and duration of RAAS inhibitor treatment and RAAS inhibitor side effects were not recorded. Although we performed an additional analysis after patients with discontinuation and changes of RAAS inhibitors during the first 12 months after CABG, prescription of RAAS inhibitors from outside the clinic may have been missed. Despite these limitations, our results provide valuable information for clinicians regarding the use of RAAS inhibitors for secondary prevention after CABG.

## Conclusions

In CABG patients, ARBs may have comparable protective effects to ACEi and be a reasonable alternative for intolerant patients. The beneficial effects of ARBs depending on sex and follow-up period require further investigation.

## Methods

### Study population

The study protocol was approved by the Institutional Review Board of Samsung Medical Center (IRB No. 2018-10-105) and conducted according to the guidelines of the Declaration of Helsinki. Considering that this was a retrospective study and risk to patients was therefore minimal, the need for individual consent was waived by the Institutional Review Board. From January 2001 to January 2015, 5453 consecutive adult patients who underwent CABG at Samsung Medical Center (Seoul, Korea) were initially enrolled. Inclusion criteria were patients who were prescribed for ACEi or ARBs at discharge after CABG, and the patients were classified into the ACEi or ARB group. The patients who were prescribed for alternative types of medication afterward were analyzed according to the intention-to-treat approach.

### Data collection

We used Clinical Data Warehouse Darwin-C, which is an electric system designed to search for and collect data from electronic medical record systems, to extract all adult CABG patients and their prescriptions at discharge. After finalizing the list of the patients for the study, prescriptions at follow-up visits to the outpatient department were collected in the same manner to identify discontinuation of RAAS inhibitors, conversion to other types of RAAS inhibitors or replacement by other antihypertensives. Baseline characteristics were extracted automatically from electronic medical records with the aid of the institutional medical information department and were organized using a standardized form and protocol by a single investigator who was blinded to the prescription at discharge. Death of patients was determined by searching the national database. Other postoperative clinical outcomes and causes of death were collected through the manual review of each case by other investigators who were blinded to baseline characteristics.

### Study outcomes and definitions

Clinical outcomes were defined as stated in a report on cardiovascular events in clinical trials by the ACC/AHA (American College of Cardiology Foundation/American Heart Association task force)^26^. The primary outcome was a MACCE. It is the composite of all-cause death, cardiac death, MI, repeat revascularization, and stroke during a median follow-up period of 48 months. Secondary outcomes included each composite of MACCE, admission for HF, and graft failure. Incidences of outcomes within 12 months were also compared. MI was defined according to the fourth universal definition^27^. Stroke was defined as a cerebrovascular accident which included both ischemic and hemorrhagic events with neurologic symptoms lasting at least 24 hours. Admission for HF was defined as a need for in-hospital treatment for at least 24 hours because of symptoms or objective evidence of new or worsening HF.

### Statistical analysis

The significance of differences in continuous variables between groups was assessed using the Mann-Whitney’s U-test if applicable. The Shapiro-Wilk test was used as a normality test. Chi-square or Fisher’s exact tests were used for categorical variables. We selected covariates with a p-value under 0.05 and those that seemed to be clinically relevant. We included these covariates into multivariate Cox’s regression analysis to adjust for the effects of confounding factors. The followings were covariates for adjustment: age, sex, hypertension, diabetes mellitus, chronic kidney disease, peripheral artery occlusive disease, left ventricular ejection fraction under 40%, off-pump CABG, history of old MI, and beta-blocker prescription. We also estimated HRs and 95% CIs with clinical relevance or with a p-value <0.05. Survival curves were constructed using Kaplan-Meier estimates and compared by the log-rank test.

We used propensity score matching to generate a matched population to minimize the effects of potential confounding factors and selection bias. We assumed that covariates were well balanced when the absolute standardized mean difference between the matched groups was less than 0.1. In the propensity-matched population, we compared continuous variables with a paired t-test and categorical variables with a stratified chi-square test. We compared HRs for outcomes using univariate Cox’s proportion hazard regression models in the matched population with or without Firth’s penalized likelihood approach.

In addition to intention-to-treat analysis, we performed analyses in the population after excluding patients with changes of RAAS inhibitor use during the 12 months after CABG. We also performed a subgroup analysis using Cox’s regression model to find any hidden interactions between risk factors and RAAS inhibitor use. All statistical analyses were performed with SAS 9.4 (SAS Institute Inc., Cary, NC, USA) and SPSS version 20 (SPSS, Inc., Chicago, IL, USA). All tests were two-tailed and assumed to be statistically significant if the p-value was less than 0.05. Continuous variables are presented as means ± standard deviations and categorical variables are expressed as percentages.

## Supplementary Information

Supplementary Information.

Supplementary Information 2.
