# Supplementary material for: Comparative Efficacy of Angiotensin Converting Enzyme Inhibitors and Angiotensin Receptor Blockers after Coronary Artery Bypass Grafting
Source: Sci Rep. 2020 Feb 3;10:1716. doi: 10.1038/s41598-020-58705-0 (PMC6997426; doi:10.1038/s41598-020-58705-0)
Supplement: Supplementary file 1 — Supplementary Information. [file 41598_2020_58705_MOESM1_ESM.docx]

**Comparative Efficacy of Angiotensin Converting Enzyme Inhibitors and Angiotensin Receptor Blockers after Coronary Artery Bypass Grafting**

Jeayoun Kim^1^, Jungchan Park^1^, Jong-Hwan Lee^1^, Jeong Jin Min^1^, Seung-Hwa Lee^2*^, Young Tak Lee^3^, Wook Sung Kim^3^, Sanghoon Song^4^, Jung Hyun Yeo^1^, and Hyojin Cho^1^

^1^Department of Anesthesiology and Pain Medicine, Samsung Medical Center, Sungkyunkwan University School of Medicine, Seoul, Korea

^2^Division of Cardiology, Department of Medicine, Heart Vascular Stroke Institute, Samsung Medical Center, Sungkyunkwan University School of Medicine, Seoul, Korea

^3^Department of Thoracic and Cardiovascular Surgery, Samsung Medical Center, Sungkyunkwan University School of Medicine, Seoul, Korea

^4^Department of Anesthesiology and Pain Medicine, Soonchunhyang University Seoul Hospital, Seoul, Korea

**Supplementary Table 1.** Incidence Rate and Hazard Ratio of the Population Without Discontinuation or Changing the Class of RAAS inhibitor

| **N, (%)** | **ARB group (N = 288)** | **ACEi group (N = 781)** | **Hazard Ratio (95% CI)** | **p-value** |
| --- | --- | --- | --- | --- |
| *4-year follow up* |  |  |  |  |
| MACCE | 28 (9.6) | 94 (11.9) | 0.66 (0.42-1.03) | 0.07 |
| total death | 11 (3.8) | 24 (3.0) | 1.15 (0.52-2.53) | 0.73 |
| cardiac death | 4 (1.4) | 7 (0.9) | 1.61 (0.39-6.59) | 0.51 |
| MI | 5 (1.7) | 11 (1.4) | 0.73 (0.22-2.36) | 0.59 |
| re-revascularization | 7 (2.4) | 35 (4.4) | 0.55 (0.24-1.27) | 0.16 |
| Stroke | 10 (3.4) | 35 (4.4) | 0.55 (0.26-1.16) | 0.11 |
| Values are n (%)  RAAS inhibitor, Renin-angiotensin-aldosterone system inhibitor; ARB, Angiotensin receptor blocker; ACEi, Angiotensin converting enzyme inhibitor; CI, Confidence interval; MACCE, Major adverse cardiovascular and cerebrovascular events and composite of total death, cardiac death, myocardial infarction, re-revascularization and stroke; MI, Myocardial infarction  Cox hazard model regression analysis was adjusted for Age, Sex, diabetes mellitus, hypertension, left ventricular ejection fraction<40%, chronic renal failure, peripheral artery disease, old myocardial infarction, beta-blocker therapy, off-pump coronary artery bypass grafting | | | | |
