# Supplementary material for: Comparative Efficacy of Angiotensin Converting Enzyme Inhibitors and Angiotensin Receptor Blockers after Coronary Artery Bypass Grafting
Source: Sci Rep. 2020 Feb 3;10:1716. doi: 10.1038/s41598-020-58705-0 (PMC6997426; doi:10.1038/s41598-020-58705-0)
Supplement: Supplementary file 2 — Supplementary Information 2. [file 41598_2020_58705_MOESM2_ESM.docx]

**Comparative Efficacy of Angiotensin Converting Enzyme Inhibitors and Angiotensin Receptor Blockers after Coronary Artery Bypass Grafting**

Jeayoun Kim^1^, Jungchan Park^1^, Jong-Hwan Lee^1^, Jeong Jin Min^1^, Seung-Hwa Lee^2*^, Young Tak Lee^3^, Wook Sung Kim^3^, Sanghoon Song^4^, Jung Hyun Yeo^1^, and Hyojin Cho^1^

^1^Department of Anesthesiology and Pain Medicine, Samsung Medical Center, Sungkyunkwan University School of Medicine, Seoul, Korea

^2^Division of Cardiology, Department of Medicine, Heart Vascular Stroke Institute, Samsung Medical Center, Sungkyunkwan University School of Medicine, Seoul, Korea

^3^Department of Thoracic and Cardiovascular Surgery, Samsung Medical Center, Sungkyunkwan University School of Medicine, Seoul, Korea

^4^Department of Anesthesiology and Pain Medicine, Soonchunhyang University Seoul Hospital, Seoul, Korea

**Supplementary Table 2.** The numbers of MACCE and cancer per 1000 months

|  | **ARB group** | **ACEi group** |
| --- | --- | --- |
|  | **(N=298)** | **(N=900)** |
| MACCE |  |  |
| Events | 31 | 8 |
| Total person-month | 29657.1 | 9111.6 |
| Per 1000 Months | 1.045281 | 0.878002 |
| Cancer |  |  |
| Events | 101 | 29 |
| Total person-month | 27841.9 | 8923.2 |
| Per 1000 Months | 3.627626 | 3.249955 |
| Values are n (%)  MACCE, Major adverse cardiovascular and cerebrovascular events and composite of total death, cardiac death, myocardial infarction, re-revascularization and stroke; ARB; Angiotensin receptor blocker; ACEi, Angiotensin converting enzyme inhibitor | | |
